# Tropomyosin, the major tropical oyster *Crassostrea belcheri* allergen and effect of cooking on its allergenicity

**DOI:** 10.1186/s13223-015-0099-4

**Published:** 2015-10-26

**Authors:** Zailatul Hani Mohamad Yadzir, Rosmilah Misnan, Faizal Bakhtiar, Noormalin Abdullah, Shahnaz Murad

**Affiliations:** Allergy and Immunology Research Centre, Institute for Medical Research, Jalan Pahang, 50588 Kuala Lumpur, Malaysia; Department of Biology, Faculty of Science and Mathematics, Universiti Pendidikan Sultan Idris, 35900 Tanjong Malim, Perak Malaysia

**Keywords:** Tropical oyster, *Crassostrea belcheri*, IgE reactivity, Tropomyosin, Major allergen, Immunoblot, Cooking processing

## Abstract

**Background:**

Many types of shellfish including oysters are sometime cooked before ingestion and it has been demonstrated that cooking may affect the allergenicity of food. Therefore, the aim of our present study is to identify major and minor allergens of tropical oyster (*Crassostrea belcheri*) and to investigate the effect of different cooking processing on the allergenicity of this oyster.

**Methods:**

Raw, boiled, fried and roasted extracts of oyster were prepared. Protein profiles were analysed using sodium dodecyl sulphate–polyacrylamide gel electrophoresis (SDS-PAGE). Major and minor allergens and allergenicity patterns of all extracts were then determined by immunoblotting with sera from patients with positive skin prick tests (SPT) to the raw oyster extract. Mass-spectrometry was used to identify the major allergenic proteins of this oyster.

**Results:**

SDS-PAGE of the raw extract showed 15 protein bands (20–180 kDa). In contrast, smaller numbers of protein bands were demonstrated in the boiled extract, those ranging between 40–42 and 55–150 kDa were denatured, whereas the protein profiles were altered to a similar degree by frying or roasting. The 37 kDa proteins had the highest frequency of IgE-binding (95 %), thus identified as the major allergen of this tropical oyster. Other minor IgE-binding proteins were observed at various molecular weights. Immunoblot of raw extract yielded 11 IgE-binding proteins. The cooked extracts showed only a single IgE-binding protein at 37 kDa. Mass spectrometry analysis of the 37 kDa major allergen identified this spot as tropomyosin.

**Conclusions:**

Cooked extracts produce lower IgE-binding than raw extract, which suggest that thermal treatment can be used as a tool in attempting to reduce oyster allergenicity by reducing the number of IgE-reactive bands. The degree of allergenicity of this oyster was demonstrated in the order raw > boiled > fried ≈ roasted. A heat-resistent 37 kDa protein, corresponding to tropomyosin, was identified as the major allergen of this tropical oyster.

## Background

Oysters are naturally one of the most nutritionally well balanced of foods [1]. They are low in fat, calories and cholesterol in addition to being high in protein, iron, omega 3 fatty acids, calcium, zinc and vitamin C [1]. Unfortunately, oysters are also one of an elicitors of IgE-mediated type I allergy [2–4].

It is well documented that the ingestion of oyster, skin and mucosal contact and the inhalation of aerosolized oyster proteins during cooking or in an occupational setting can cause a large variety of clinical symptoms in sensitized patients, such as urticaria, angioedema, atopic dermatitis, asthma, rhinitis, vomiting, diarrhea and anaphylaxis [2–4].

Currently, the only effective treatment to prevent oyster-induced allergies is a specific avoidance diet [5]. However, avoidance is often difficult due to unintentional cross-contamination or the addition of oyster as ingredients in some common sauces and condiments [5].

The identification of allergenic proteins in a particular species is an important step for the development of more accurate allergy tests and for the definition of more effective management of patients [6–8]. Until now, tropomyosin was the only well recognized allergen in oyster [9, 10]. Tropomyosins are highly conserved proteins with a typical coiled-coil structure that are necessary for regulating muscle contraction [11–13]. The highly conserved amino acid sequence of tropomyosin is responsible for its identification as a panallergen for cross-reactivity between crustaceans, molluscs, insects and arachnids [11, 14–23].

Oyster may be consumed raw or cooked. Cooked oysters are usually subjected to some form of heat treatment including boiling, frying or roasting to enhance texture and flavours or to ensure microbiological safety. At the same time, heating can alter proteins by inducing denaturation (loss in tertiary and/or secondary interactions), formation of new intra- or inter-molecular bonds, aggregation, and/or rearrangements of disulfide bonds, as well as other conformational modifications, which can ultimately lead to changes in allergen reactivity [24]. In some cases, thermal treatment has been shown to decrease allergen reactivity [24–26], yet in other cases, thermal treatment increases allergen potency [27–29], possibly by exposing new IgE-binding sites. Therefore, understanding thermal treatment seems to be important in oyster allergenicity.

*Crassostrea belcheri* (tropical oyster) is a commonly consumed oyster in Malaysia. Our previous study indicated that it has provoked allergenic reactions in some individuals [30], but there has been no study about the allergenic proteins of this local oyster. The aim of this study was to identify the major allergenic proteins of the tropical oyster and to investigate the effect of different cooking methods on the allergenicity of this oyster.

## Methods

### Preparation of tropical oyster extracts

For the preparation of the raw oyster (*Crassostrea belcheri*) extract, the inner muscle tissue was homogenized in phosphate buffered saline (PBS), pH 7.2 and extracted overnight at 4 °C, followed by centrifugation at 4500 and 14,000 rpm, for 30 and 15 min, respectively. The clear supernatant was then recovered and sterilized by passing through a 0.22 μm syringe filter, frozen and lyophilized. The lyophilized extracts were stored at −20 °C until use. The boiled oyster extract was prepared by boiling muscle tissue for 10 min at 100 °C, the fried oyster extract was carried out by frying muscle tissue with vegetable oil for 10 min and the roasted oyster extract was prepared by roasting at 180 °C for 10 min followed by homogenization according to the same protocol as above. Protein concentrations of the extracts were determined using the total protein kit (Sigma, USA), according to the manufacturer’s instructions. Bovine serum albumin (BSA) was used as the protein standard.

### Human sera

Sera of 20 patients with a history of oyster allergy and a positive skin prick test (SPT) to raw oyster extract were used in this study. This study was approved by the Medical Research and Ethics Committee (MREC), Ministry of Health Malaysia and informed consent was obtained from the patients or their relatives.

### Sodium dodecyl sulfate polyacrylamide-gel electrophoresis (SDS-PAGE), two dimensional-gel electrophoresis (2-DE) and immunoblotting

SDS-PAGE and 2-DE were carried out as described previously [15, 16]. The components of extracts from oyster that were separated by gel electrophoresis and recognized by IgE antibodies in sera were then analyzed by immunoblotting [15, 16].

### Mass spectrometry analysis

The Coomassie-stained protein spots corresponding to those recognized by the above sera were manually excised and transferred to microcentrifuge tubes. These protein spots were analyzed using mass spectrometry analysis by First Base Laboratories Sdn Bhd, Malaysia. Protein samples were trypsin digested and peptides extracted according to standard techniques. Peptides were analyzed by matrix-assisted laser desorption-ionization time of flight (MALDI-TOF) mass spectrometer using a 4800 Proteomics Analyzer. Spectra were analyzed to identify proteins of interest using Mascot sequence matching software (Matrix Science) with Ludwig NR Database and taxonomy set to other metazoa.

## Results

### Oyster protein profiles and IgE-binding proteins

**Fig. 1:**
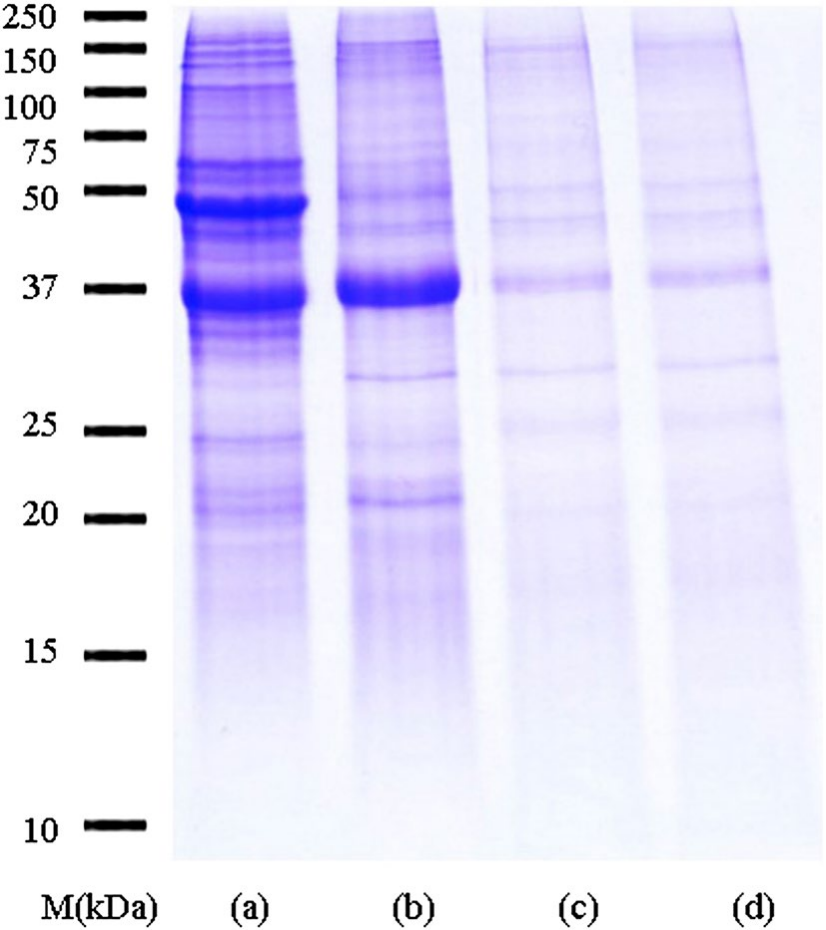
SDS-PAGE profiles of raw (**a**), boiled (**b**), fried (**c**) and roasted (**d**) extracts of *Crassostrea belcheri* (tropical oyster). *Lane M* molecular mass markers

SDS-PAGE of raw oyster extract demonstrated 15 protein bands in the range of 20–180 kDa. Fewer bands were detected in the boiled extract as several protein bands between 40–42 and 55–150 kDa were sensitive to heat and thus were no longer detected in the gel. On the other hand, most of the protein bands in fried and roasted extracts were completely denatured. Therefore, they were identified as heat-sensitive proteins. In contrast, a 37 kDa protein was very stable to heating and it can be detected in all oyster extracts even after boiling, frying or roasting (Fig. 1).

**Fig. 2:**
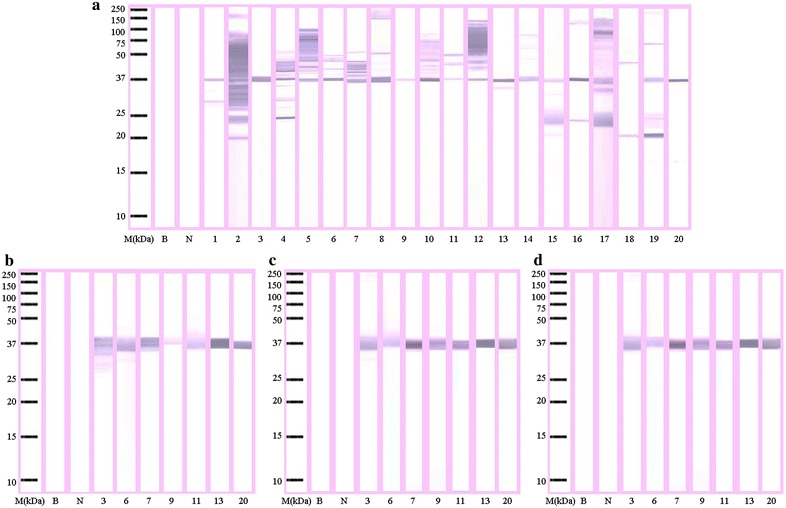
Immunoblotting of raw (**a**), boiled (**b**), fried (**c**) and roasted (**d**) extracts of *Crassostrea belcheri* (tropical oyster). *Lane M* molecular mass markers, *lanes 1*–*20* immunoblots showing binding of IgE from different serum samples, *lane N* immunoblot using serum from a non-allergic individual, and *lane B* blank

Twelve components with molecular weights ranging from about 20–180 kDa showed IgE-binding activity in the raw oyster extract. Regarding IgE reactivity, each patient had different patterns of IgE recognition (1–12 IgE-binding bands). The component with a molecular weight of about 37 kDa showed the highest frequency of IgE-binding (95 %) and was identified as the major allergen for this oyster (Fig. 2a). IgE-binding proteins were also detected at 5 % (150 kDa), 10 % (33 kDa), 15 % (180, 75, 36, 30, 20 kDa), 20 % (100 kDa), 25 % (90, 65 kDa), 30 % (25 kDa), 35 % (40 kDa) and 45 % (50, 42 kDa), but only as minor allergens.

On the other hand, immunoblotting of the cooked extracts detected only one IgE-binding at 37 kDa and more staining to this band was observed in most sera tested (Fig. 2b–d). No binding was observed in the negative control and blank strip.

### Oyster 2-DE profiles and IgE-binding spots

**Fig. 3:**
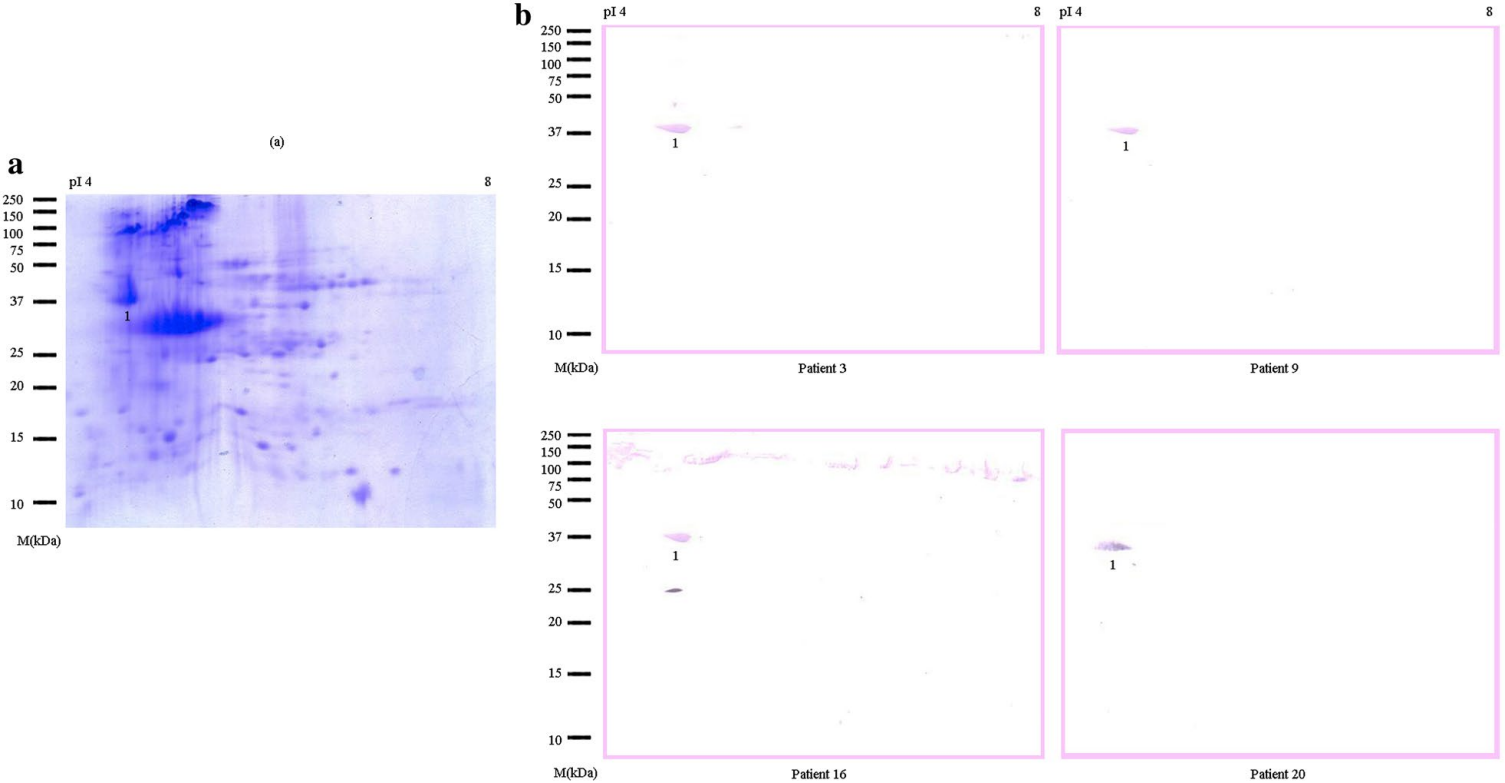
Two dimensional electrophoresis and immunoblot analysis of *Crassostrea belcheri* (tropical oyster). **a** Coomassie blue stained blot; **b** immunoblot with individual patients’ sera. Spot number 1 was sent for mass spectrometry analysis. *Lane M* molecular mass markers

Figure 3a demonstrated the 2-dimensional gel map of proteins from the raw oyster extract. About 50 spots with molecular masses from 20 to 180 kDa and isoelectric point (pI) between 4 and 8 were visible with coomassie blue staining. The simple IgE-stained patterns of the oyster protein spots obtained with four sera are shown in Fig. 3b. A highly reactive protein spot with a molecular mass of 37 kDa and pI of 4.57 was observed in all of the patients’ serum samples tested.

### Allergen identification

**Fig. 4:**
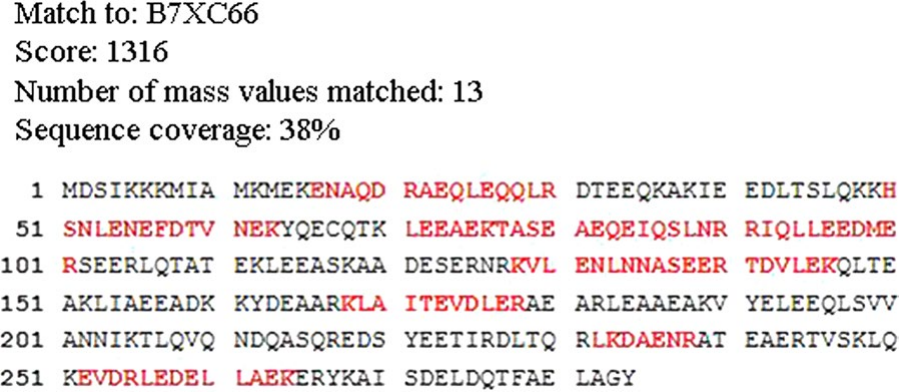
Protein spot 1 peptide mass fingerprint analysis which correlates with *Crassostrea gigas*. Matched peptides shown in *bold red*

In the peptide mass fingerprint analysis, the excised protein spot number 1 showed the highest correlation with tropomyosin from Pacific oyster *C. gigas* (accession no. B7XC66) with peptide sequence coverage of 38 %. The corresponding sequence coverage is shown in Fig. 4.

## Discussion

Cooking which can be considered a form of thermal treatment affect the allergenicity of the oyster protein. In our study, cooking processing, such as boiling, frying and roasting, produced changes on the SDS-PAGE protein profile of oyster allergens. Differences seen in these protein profiles may be attributed to temperatures at which each heat treatment was executed. Roasting and frying were performed at higher temperatures (180 and 120 °C, respectively) whereas boiling was performed at relatively lower temperatures (100 °C). Compared to the raw extracts, boiling causes a remarkable reduction in the number of protein bands between 40–42 and 55–150 kDa. Both frying and roasting showed a similar protein profile, with most of the bands clearly eliminated compared with those of the raw and boiled extracts. In contrast, a prominent heat-resistant 37 kDa protein was detected in the SDS-PAGE even after boiling, frying or roasting. Many food allergens are resistant to denaturation, which allows them to retain the ability to elicit an immune reaction under conditions such as heating and enzymatic degradation [31].

The loss of proteins in boiled, fried and roasted extracts may be related to the effects of heat on them. Thermal treatment can potentially cause protein modifications, which include protein fragmentation, denaturation, or intra- or inter-molecular protein crosslinking [32–35]. Fragmented protein sections that are smaller than the resolution limit of the gel may travel through the acrylamide pores more quickly, causing them to ultimately be lost in the buffer. Conversely, proteins that have been modified by intermolecular crosslinking (i.e., protein aggregation) are often too large to migrate through the gel, and are not clearly detectable via SDS-PAGE, but may appear congested in the wells of the gel [32, 33, 35]. However, proteins modified via covalent modification such as intramolecular crosslinking can maintain approximately the same molecular weight, yet may often migrate through polyacrylamide gels with slight difficulty, as they are not completely linearized under reducing conditions. Thus, the protein bands corresponding to the modified proteins can have a smeared appearance in immunoblotting analyses [34]. This was apparent in Fig. 2b–d, where 37 kDa bands were notably smeared in cooked extracts; therefore, it appears that thermal treatments may have caused oyster allergen modification via a putative covalent modification. This speculates that the conformational modification of the 37 kDa allergen may have changed the reactivity of the allergen by altering IgE-binding epitopes. Taheri-Kafrani et al. [33] have shown that covalent modifications, such as glycation, have been attributed to the Maillard reaction, where reducing sugars interact with proteins in a non-enzymatic browning reaction. Previous experiments using scallop tropomyosin displayed an increase in allergen potency with the progression of the Maillard reaction [36]. Kamath et al. [37] also reported that tropomyosin from oyster *C. gigas* has been shown to increase with heating as detected by monoclonal antibodies possibly due to the Maillard reaction. The effects of the Maillard reaction appear to be dependent on the sample and the amount and type of reducing sugars present, as well as many other variables, such as treatment temperature.

Allergens can be defined as being either major or minor. A major allergen is defined as one, recognized by IgE from more than 50 % of individuals sensitive to the particular food from where the allergen originate [38]. Until now, tropomyosin was the only well recognized allergen in oyster [9, 10]. Tropomyosin, with a molecular weight of about 34–38 kDa, is a group of highly conserved actin-binding protein present in muscle and non-muscle cells and plays a central role in muscle contraction [11–13]. Similarly, our study has also identified the 37 kDa major allergen as tropomyosin by mass spectrometry analysis. Our findings confirmed and strengthened earlier reports on the heat stability of tropomyosin. After heat treatment, only tropomyosin exhibited the IgE-binding capacity. Tropomyosin is heat resistant and retains its IgE-binding ability even after prolonged heating [37, 39, 40]. The ability of tropomyosin to withstand heat-treatment and most known type of food processing techniques can be attributed to its exceptionally stable alpha helical coiled-coil secondary structure [22, 38].

## Conclusions

In conclusion, thermal treatment can be used as a tool to reduce oyster allergenicity by reducing the number of allergens. A heat-resistent 37 kDa protein, corresponding to tropomyosin, was identified as the major allergen of this tropical oyster.

## Abbreviations

SPT: Skin prick test
SDS-PAGE: sodium dodecyl sulfate polyacrylamide-gel electrophoresis
2-DE: two dimensional-gel electrophoresis
